# The preventive effect of graded nursing based on perineal assessment tool on incontinence-associated dermatitis in patients with severe traumatic brain injuries

**DOI:** 10.12669/pjms.41.6.11691

**Published:** 2025-06

**Authors:** Qiuxia Yin, Liping Huang, Xiaoqin Rui, Aiqin Zhang

**Affiliations:** 1 Qiuxia Yin, Department of Nursing (ICU), Jinling Clinical Medical College, Nanjing Medical University, Nanjing, Jiangsu Province 211166, P.R. China; 2 Liping Huang, Department of ICU, Jiangyin People’s Hospital, Jiangyin, Jiangsu Province 214400, P.R. China; 3 Xiaoqin Rui, Department of Neurosurgery, Jiangyin People’s Hospital, Jiangyin, Jiangsu Province 214400, P.R. China; 4 Aiqin Zhang, Department of Medical Information Data Room, Jinling Clinical Medical College, Nanjing Medical University (General Hospital of Eastern Theater Command), Nanjing, Jiangsu Province 210002, P.R. China

**Keywords:** Graded nursing, Incontinence associated dermatitis, Perineal Assessment Tool, Traumatic brain injury

## Abstract

**Objective:**

Severe traumatic brain injury (TBI) is associated with substantial central nervous system damage, making the patients prone to urinary or fecal incontinence and incontinence-associated dermatitis (IAD). This study aimed to evaluate the clinical value of graded nursing based on the Perineal Assessment Tool (PAT) for preventing and treating IAD in patients with severe TBI.

**Methods:**

This retrospective analysis was conducted at Jiangyin People’s Hospital and included prospectively collected data from 189 patients with severe TBI admitted to the intensive care unit from March 2022 to December 2023. Among them, 94 patients received traditional nursing (traditional group), and 95 received graded nursing based on the PAT risk assessment tool (graded nursing group). The PAT score, IAD incidence, quality of life, and nursing satisfaction of patients were analyzed before and after intervention.

**Results:**

After intervention, the PAT and quality of life (QOL) scores in both groups increased compared to before intervention and were higher in the grading nursing group compared to the traditional group (*P*<0.05). The incidence and duration of IAD in the graded nursing group were lower, while the nursing satisfaction was higher compared to the traditional group (*P*<0.05).

**Conclusions:**

Compared with traditional nursing, graded nursing based on PAT in severe TBI patients can reduce the incidence of IAD, improve QOL, and increase patient satisfaction.

## INTRODUCTION

Severe traumatic brain injury (TBI) is associated with significant damage to the central nervous system, and patients are often in a coma and experience urinary and fecal incontinence, which may eventually lead to incontinence-associated dermatitis (IAD).^1^-^4^ IAD is an inflammation of the skin in the perineal area resulting from fecal and urinary incontinence, and research has shown that the incidence rate of IAD in patients with severe TBI can reach up to 35.07%.^2^,^3^ IAD is often accompanied by skin itching, papules, peeling, edema, erythema, etc.^3^,^4^ which exacerbates physical and mental discomfort and may cause bacterial infections associated with a substantial burden on the patient and the nursing personnel.^4^,^5^ Therefore, effective nursing interventions for patients with severe TBI to prevent IAD is crucial.

Traditional nursing care for TBI patients mainly involves clearing excrement and administering skin protectants locally.^6^ While such an approach can alleviate patients’ clinical symptoms to a certain extent, the overall effect is not ideal.^5^,^6^ The Perineal Assessment Tool (PAT) was specifically developed to address the problem of perineal skin injury by assessing the duration and intensity of irritation, perineal skin condition, and contributing factors that may cause diarrhea.^7^ Several recent studies have described a nursing approach that uses PAT to evaluate patients’ risk of developing IAD and construct a corresponding graded nursing plan based on the evaluation results to reduce the risk of developing IAD.^8^,^9^

Currently, there are very few reports on the effectiveness of graded nursing based on PAT in preventing IAD in patients with severe TBI. This study aimed to evaluate the effectiveness of graded nursing based on PAT for this group of patients and provide reference opinions for relevant clinical workers to prevent the occurrence of IAD in patients with severe TBI.

## METHODS

This retrospective study used prospectively collected clinical data of 189 patients with severe TBI admitted to the intensive care unit of the Jiangyin People’s Hospital from March 2022 to December 2023 for the retrospective analysis. Of them, 94 patients received traditional nursing care (traditional group), and 95 received graded nursing based on PAT (graded group).

### Ethical Approval:

The ethics committee of our hospital approved this study with the number: 2021-050; Date: October 22, 2021.

### Inclusion criteria:

- Met the diagnostic criteria for severe TBI;^1^
- Urinary and/or defecation incontinence;
- Age ≥ 18 years old;
- Complete clinical data.

### Exclusion criteria:

- Patients with a history of IAD;
- Patients with damage to the perineum and perianal skin;
- Patients with other skin diseases;
- Patients with unstable vital signs and severe heart, lung, liver, kidney, and other important organ failure;
- Patients with abnormal coagulation function.

### Traditional nursing:

The condition of the patient’s perianal and perineal skin was assessed daily. Excrements were timely and gently removed with warm water. After cleaning the area, Sanyrene (URGO, France) was applied to protect the skin. Bed sheets were kept flat, dry, and clean, and non-breathable urine pads were avoided. If the bed sheets were contaminated or damp, they were immediately replaced.

### Graded nursing based on PAT:

- *1) Construction of an intervention team:* An intervention team was created and included two incontinence nursing experts and two neurosurgery nursing experts. Specific IAD-prevention training was provided to the team members, including identification, staging, assessment, graded care, and IAD protection procedures. An easy-to-remember IAD protection knowledge card was generated based on the content of the “Interpretation of domestic and foreign expert consensuses of Incontinence-Associated Dermatitis nursing”.^10^ The knowledge card emphasized four main principles of IAD care: cleaning the skin with clean water, using single-layer diapers with good water absorption properties, maintaining ventilation and replacing damp diapers promptly, and using skin protectants and isolating irritants.
- *2) IAD risk level assessment:* It was assessed using PAT. PAT includes four determinants of perineal skin breakdown: irritant type, perineal skin condition, duration of irritation, and influencing factors (antibiotic use, hypoalbuminemia, nasal feeding, and others). Each dimension is scored 1-3 points based on the degree of risk, and the scores for each dimension are summarized. Risk classification is based on the total score, divided into low-risk (4-6 points) and high-risk (7-12 points). The assessment for all patients was completed within two hours of admission. An “IAD Protection” sign was placed at the patient’s bedside, with blue indicating low risk, yellow indicating high risk, and red indicating already occurred IAD. A detailed record of PAT score, infiltration or redness site, and range, was collected for all patients.
- *3) Graded nursing:* It was implemented based on the PAT results. For low-risk patients with IAD, nursing staff conducted a daily assessment. In every case of condition change, an evaluation was conducted immediately. All personnel received a detailed education and guidance on IAD, informing about the hazards of IAD and the necessity and clinical significance of early prevention. The skin cleaned with disposable soft, non-woven fabric soaked in warm water at 37° to 40°, air dried naturally or by gently patting dry with an absorbent towel. Skin lotion was applied on areas where patients are prone to infiltration to protect the skin. Disposable diapers with a good water absorption effect were used, and dampness was checked every two hours. Diapers were changed frequently to prevent sweating and other factors that may cause local moisture and lack of ventilation.

For high-risk patients with IAD, nursing staff conducted one assessment per shift. The local skin was cleaned and let air dry naturally. Stoma powder (Hollister) was spread evenly on the perineum with a sterile cotton swab; 3M Cavilon liquid dressing was sprayed 10-15 cm from the skin. For the folds on the buttocks and thighs, the folds were gently separated before spraying 3-4 times a day. Urinary catheterization was provided for individuals with urinary incontinence. Drainage devices, sanitary tampons, and built-in anal canals were provided to individuals with watery stools. External ostomy bags were used for pasty stools. For patients with watery stools and diarrhea occurring ≥ 4 times, care measures for skin integrity, pain, and diarrhea were taken based on specific conditions.

The nursing was provided to the patients for two weeks and the scores were measured after intervention.

### Collected indicators:

- Basic clinical characteristics of patients, including gender, age, body mass index (BMI), type of incontinence, frequency of incontinence, and stool characteristics.
- The incidence of IAD during intensive care unit (ICU) stay. Mild: No blisters or dryness on the skin, with some areas showing redness or erythema; Moderate: redness, swelling, and rash on the skin, with local damage, exudation, and bleeding; Severe: Skin damage and erosion.
- Duration of IAD: the time from the onset of IAD to its disappearance.
- Quality of life, assessed using the Short Form Health Survey (SF-36). The survey evaluates social function, physical pain, health status, and mental state, scoring 0-100 points for each dimension. A higher score indicates a better quality of life.
- Nursing satisfaction. The Newcastle Satisfaction with Nursing Scale (NSNS) was used to evaluate patients’ nursing satisfaction, consisting of five dimensions and 19 items. Using the Likert 5-point scoring method, 1-5 points were assigned respectively, and the total score was calculated on a percentage scale. A higher score indicates higher patient satisfaction with the nursing work.

### Statistical analysis:

All data analyses were conducted using SPSS 26.0 software (IBM Corp, Armonk, NY, USA). The Shapiro-Wilk test was used to evaluate the normality of the data. Normally distributed data were represented by mean ± standard deviation, an independent sample t-test was used for inter-group comparison, and a paired t-test was used for intra-group comparison before and after the intervention. The count data were represented by the number of cases and compared using the Chi-square test. PRISM8.0 software (GraphPad, San Diego, USA) was used to draw box plots of SF-36 score, and nursing satisfaction before and after intervention. When *P*<0.05, the difference was considered statistically significant.

## RESULTS

This study retrospectively analyzed the clinical data of 189 patients (116 males and 73 females) with severe TBI. The age ranged from 29 to 85 years, with a mean age of 55.4 ± 10.1 years. There were 95 patients in the graded nursing group and 94 in the traditional group, with no significant difference in the basic clinical characteristics between the two groups (*P*>0.05) (Table-I).

**Table-I:** Comparison of basic clinical characteristics between two groups.

Characteristics	Graded nursing (n=95)	Traditional Group (n=94)	t/χ^2^	P
Male (yes), n (%)	53 (55.8)	63 (67.0)	2.514	0.113
Age (years), mean±SD	56.6±10.4	54.2±9.7	1.616	0.108
BMI (kg/m²), mean±SD	23.4±2.7	23.8±3.1	-1.109	0.269
Type of incontinence, n (%)				
Fecal incontinence	13 (13.7)	19 (20.2)	1.555	0.460
Urinary incontinence	16 (16.8)	13 (13.8)		
Fecal and Urinary incontinence	66 (69.5)	62 (66.0)		
Incontinence frequency (times/day), n (%)				
<3	18 (18.9)	12 (12.8)	1.392	0.499
3~6	16 (16.8)	16 (17.0)		
>6	61 (64.3)	66 (70.2)		
Stool property, n (%)				
Soft stool	15 (14.8)	11 (11.7)	1.225	0.542
Diluted stool	19 (20.0)	24 (25.5)		
Watery stool	61 (64.2)	59 (62.8)		

***Note:*** BMI, Body Mass Index.

**Fig.1:**
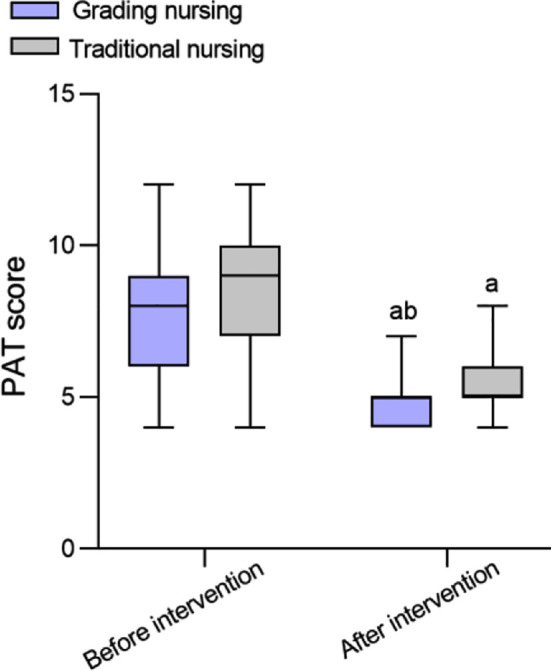
Comparison of PAT scores between two groups. Compared with before treatment in the same group, ^a^*P*<0.05; Compared with the traditional group, ^b^*P*<0.05. PAT, Perineal Assessment Tool.

As shown in Fig.1, there was no significant difference in the PAT scores between the two groups before intervention (*P*>0.05). After intervention, the PAT scores of both groups decreased and were significantly lower in the grading nursing group compared to the traditional group (*P*<0.05). During ICU stay, the incidence of IAD in the graded nursing group (6.3%) was lower than that in the traditional group (18.1%) (*P*<0.05). Similarly, the duration of IAD in the graded group was lower than that in the traditional group (*P*<0.05) (Table-II).

**Table-II:** Comparison of IAD incidence and duration between two groups.

Group	n	Severity of IAD, n(%)				IAD duration (day)
		Mild	Moderate	Severe	Total incidence rate	
Grading Group	95	4 (4.2)	2 (2.1)	0 (0.0)	6 (6.3)	3.2±1.2
Traditional group	94	8 (8.5)	7 (7.4)	2 (2.1)	17 (18.1)	5.1±1.5
*χ^2/ t^*					6.123	-2.825
*P*					0.013	0.010

**Fig.2:**
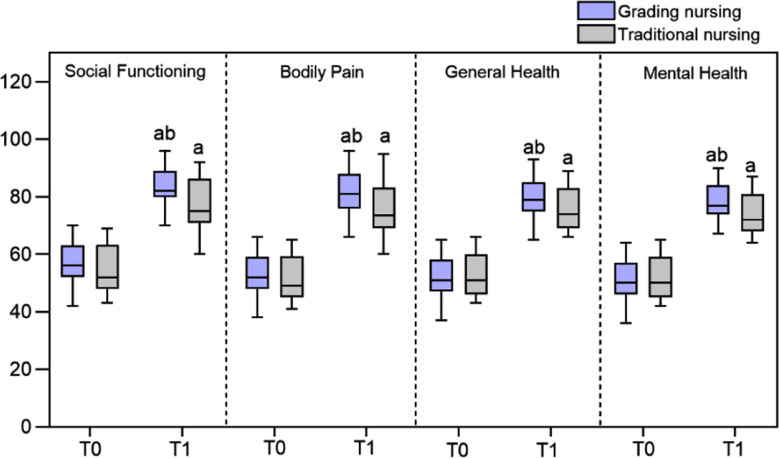
Comparison of quality of life between two groups; Compared with before treatment in the same group, ^a^*P*<0.05; Compared with the traditional group, ^b^*P*<0.05.

**Fig.3:**
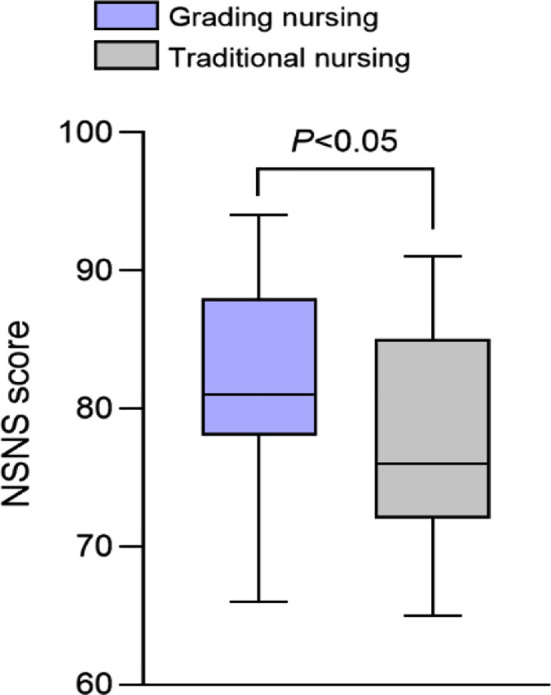
Comparison of NSNS scores for nursing satisfaction between two groups. ***NSNS:*** Newcastle Satisfaction with nursing scale.

As summarized in Fig.2, the scores of social function, bodily pain, general health, and mental health were comparable in the two groups before intervention (*P*>0.05). Postintervention scores were markedly increased in the two groups compared to preintervention values and were considerably higher in the grading nursing group compared to the traditional group (*P*<0.05). Fig.3 shows that the nursing satisfaction NSNS score of the graded group is higher than that of the traditional group (*P*<0.05).

## DISCUSSION

The results of this study show that compared with traditional nursing interventions, graded nursing based on PAT is more effective in preventing IAD in patients with severe TBI. The research hotspots of IAD management mainly focus on constructing structured skin care plans and establishing nursing processes for preventing IAD.^11^-^13^ The traditional nursing approach does not consider patients’ risk factors, making recognizing the different risk levels difficult.^13^,^14^ Therefore, the evaluation and grading of nursing care need to be systematized to provide clinical nurses with evidence-based support and facilitate implementation.^12^-^14^ The PAT is an effective tool specifically designed to assess IAD risks.^8^,^9^,^15^ It can comprehensively evaluate the risk of developing IAD in patients from multiple dimensions, such as skin condition, incontinence, and mobility.^15^,^16^ Since the physical functions of patients with severe TBI are unique,^17^,^18^ implementing graded nursing based on PAT results reflects the personalization and pertinence of nursing.^8^,^19^ Wang et al.^20^ found that such an approach in patients with different risk levels receiving varying degrees and types of nursing interventions can allocate nursing resources reasonably. This ensures that more energy and resources are invested in the patients who need them the most, improving the efficiency and quality of care.

In this study, the PAT scores of both groups decreased after the intervention compared to before the intervention, and the grading nursing based on PAT was associated with a lower PAT score than the traditional approach. The graded nursing based on PAT can adjust nursing strategies more carefully according to patients’ specific situation, avoiding further skin damage caused by IAD.^19^,^21^ Timely classification of PAT risk in patients with severe TBI and implementation of IAD grading prevention measures based on the level of risk may prevent the damage of irritants such as urination and defecation to the skin while enhancing the protective function of the skin barrier.^21^,^22^

This study showed that compared with the traditional group, the incidence and duration of IAD in the graded nursing group were significantly lower. Graded nursing intervention based on PAT allows a comprehensive prediction and identification of high-risk patients based on a full understanding of the individual challenges of severe TBI. This approach facilitates the implementation of hierarchical dynamic management and the adoption of personalized and precise nursing measures to prevent the development of IAD based on its different levels. Early detection and treatment are beneficial for promoting the improvement of dermatitis and shortening the duration of IAD.^20^-^22^ Moreover, reducing the incidence of IAD is of great significance for the rehabilitation of patients with severe TBI. It lowers the risk of IAD-associated complications, preventing further aggravation of the patient’s condition.^19^,^21^,^22^

This study showed that while the quality of life and NSNS scores of both groups increased, the quality of life of patients who received graded nursing based on the PAT was higher than that of the traditional group. Graded nursing based on PAT not only focuses on symptom relief but also covers multiple aspects of assessment, allowing the nursing staff to comprehensively evaluate the health status of patients with severe TBI. The evaluation results may then be used to provide targeted care according to specific health problems.^21^ A graded care approach will provide personalized nutritional support for patients with nutritional risks and ensure that they consume sufficient nutrients, which will help with physical recovery and improve their health status.^23^ Additionally, the nursing staff may provide tailored rehabilitation guidance, which will help to facilitate the patient’s physical function recovery and enhance immunity, further improving satisfaction with nursing care.^24^

Risk assessment is an important measure to gauge the risk of IAD in elderly patients. Nurses and staff prioritize this activity and recognize its importance in identifying IAD risks.^19^,^23^ The graded nursing based on PAT focuses on the personalized needs of patients, and nursing staff can provide more attentive services according to the patient’s risk level and specific situation. For example, in terms of ward environment management, this approach may provide a cleaner and more comfortable environment for high-risk patients to reduce sources of infection. Regarding nursing timeliness, prompt management of incontinence and skin problems can enable patients to experience higher-quality nursing services, thereby improving nursing satisfaction.^23^,^24^

### Limitations

Firstly, this study is a single-center retrospective analysis with a small sample size. Secondly, due to the influence of nurses’ clinical recognition ability, there may be certain biases in the PAT risk assessment of patients. Thirdly, the impact of graded nursing based on PAT on the recovery of other patient functions has not been analyzed. Finally, different regions and climates also have a certain effect on the occurrence of IAD. Subsequent multicenter studies are needed to validate the conclusions of this study.

## CONCLUSION

Implementing graded nursing interventions based on PAT for patients with severe TBI can reduce the incidence of IAD, improve their quality of life, and achieve high patient satisfaction. Currently, only a few studies are confirming the efficacy of graded nursing based on PAT in the prevention of IAD in patients with severe TBI. However, while this study fills the relevant gap, further improvement is needed to refine the PAT to better adapt to the needs of different patient groups in clinical practice. The nursing personnel would benefit from further strengthening the training and practice of graded nursing based on PAT and continuously improving the quality of nursing to address the nursing challenges of severe TBI patients and promote their recovery and health.

### Authors’ contributions:

**QY:** Study design, literature search and manuscript writing.

**LH, XR and AZ:** Data collection, data analysis, interpretation and critical review.

**QY:** Critical review, manuscript revision and validation.

All authors have read, approved the final manuscript and are responsible for the integrity of the study.
